# Fibrotic Phenotype of Peritumour Mesenteric Adipose Tissue in Human Colon Cancer: A Potential Hallmark of Metastatic Properties

**DOI:** 10.3390/ijms22052430

**Published:** 2021-02-28

**Authors:** Maria Tabuso, Raghu Adya, Richard Stark, Kishore Gopalakrishnan, Yee Wah Tsang, Sean James, Andrew White, Adrian Fisk, Federica Dimitri, Mark Christian, Ramesh Pulendran Arasaradnam

**Affiliations:** 1 Department of Gastroenterology, University Hospitals Coventry and Warwickshire, Clifford Bridge Road, Coventry CV2 2DX, UK; R.Arasaradnam@warwick.ac.uk; 2 Warwick Medical School, University of Warwick, Coventry CV4 7AL, UK; R.Adya@warwick.ac.uk; 3 School of Life Sciences, University of Warwick, Coventry CV4 7AL, UK; R.Stark@warwick.ac.uk; 4 Department of Pathology, University Hospitals Coventry and Warwickshire, Clifford Bridge Road, Coventry CV2 2DX, UK; Kishore.Gopalakrishnan@uhcw.nhs.uk (K.G.); YeeWah.tsang@uhcw.nhs.uk (Y.W.T.); 5 Department of Biomedical Sciences, University Hospital Coventry & Warwickshire, Coventry CV2 2DX, UK; sean.james@uhcw.nhs.uk (S.J.); Andrew.White@uhcw.nhs.uk (A.W.); adrian.fisk@uhcw.nhs.uk (A.F.); 6 Department of Biosciences, School of Science and Technology, Nottingham Trent University, Nottingham NG11 8NS, UK; F.Dimitri.1@warwick.ac.uk (F.D.); mark.christian@ntu.ac.uk (M.C.); 7 Faculty of Health and Life Sciences, University of Coventry, Priory Street, Coventry CV1 5BF, UK; 8 School of Health Sciences, University of Leicester, Leicester LE1 7RH, UK

**Keywords:** colon cancer, adipose tissue, extracellular matrix, tumour microenvironment

## Abstract

The impact of tumour associated stroma on cancer metastasis is an emerging field. However, cancer associated genes in peritumoral adipose tissue (pAT) in human colon cancer have not been explored. The aim of this study was to identify differentially expressed genes (DEGs) associated with cancer pathways in mesenteric pAT compared with adjacent adipose tissue. In total, nine patients with colon cancer pathological stage T2/T4 were employed in this study. DEGs were identified in 6 patients employing Nanostring PanCancer Pathway Panel and pathway enrichment analyses were performed. Differential expression of the 5 most up-regulated and 2 down regulated genes was validated with qRT-PCR. Results showed collagen type I alpha 1 chain (*COL1A1*) *p* = 0.007; secreted frizzled related protein (*SFRP2*) *p* = 0.057; fibroblast growth factor 7 (*FGF7*) not significant (ns); phospholipase A2, group IIA (*PLA2G2A*) ns; nerve growth factor receptor (*NGFR*) ns; lymphoid enhancer binding factor 1 (*LEF1*) *p* = 0.03; cadherin 1, Type 1, E-cadherin (epithelial) (*CDH1*) 0.09. Results have highlighted down-regulation of the Wingless/Integrated (Wnt) pathway in mesenteric pAT compared to distal adipose tissue. Highly upregulated genes in mesenteric pAT were involved in extracellular matrix (ECM)-receptor interactions and focal adhesion. Highly down regulated genes were involved in the cell cycle. Immunohistochemistry revealed differential distribution of COL1A1 showing maximum levels in tumour tissue and gradually decreasing in distant adipose tissue. *COL1A1* and down regulation of Wnt pathway may have a role in local invasion and distant metastasis. *COL1A1* may represent a stromal prognostic biomarker and therapeutic target in colon cancer.

## 1. Introduction

Despite notable advances in therapy, mainly focusing on cancer cell aberrant signalling pathways, colorectal cancer (CRC) is still responsible for high morbidity and mortality worldwide. Local-regional recurrence of colon cancer in patients undergoing curative resection with no lymphatic metastases is still observed [1]. Metastatic cancer cells have been observed in gastric cancer patients in the mesogastrium suggesting a fifth metastasis route other than the established four routes (direct invasion, lymphatic drainage, hematogenous invasion, and peritoneal dissemination) [2,3,4]. Metastatic cancer cells have also been detected in the mesentery of colorectum in CRC patients [5]. However, the mechanism of metastatic spread in the mesentery is unknown. Traditional basic science research has focused on cancer cell characteristics, such as tumour morphology, tumour cell of origin, tumour molecular pathway, and tumour mutation status. However, cancer cells are embedded in a local stromal environment constituted by infiltrating immune and stromal cells including adipocytes, macrophages, fibroblasts, monocytes, neutrophils, T-lymphocytes, B-lymphocytes, nerve cells, and extracellular matrix (ECM), constituting the tumour microenvironment (TME) [6]. CRC involves complex interactions between specific genetic and transcriptional alterations in epithelial cells and structural and metabolic remodelling within the TME. The critical role of the TME in cancer progression, evasion of immune surveillance, and therapeutic resistance has recently emerged [7,8].

The ECM is a dynamic structure which interacts with cells regulating differentiation, proliferation, and migration. Tumours are characterised by remodelling of ECM, known as desmoplasia, with increased inflammatory cell infiltration and fibrosis contributing to tumour progression and metastasis [9]. There is evidence, in breast cancer, suggesting that primary tumours of different metastatic potential differ in ECM composition [10].

In recent years a plethora of evidence has highlighted the importance of stromal cell-cancer cell interaction in cancer initiation, progression, neo-angiogenesis, invasion, and metastasis [11]. This complex bi-directional cross-talk leads not only to inter-tumoral heterogeneity amongst different CRC patients [12,13], but also to intra-tumoural heterogeneity within the same tumour [13]. This is mirrored in different clinical outcomes in patients with same stage of CRC.

Studies in breast and colon cancer have shown genetic reprogramming in the surrounding stroma, creating a tumour promoting micro-environment [14,15,16]. Genetic and epigenetic reprogramming in macroscopically normal mucosa, in proximity to the tumour, has been described as “field cancerisation”, harbouring signatures of early diagnosis in CRC [17,18], asserting a predisposing phenotype of non-neoplastic mucosa favourable to cancer initiation. More recently, there has been an interest in the genetic phenotype of tumour-associated stroma in breast cancer. It has been reported in breast cancer, that tumour-associated stroma undergoes gene expression changes similar to malignant epithelium, supporting cancer progression [19,20]. Cancer related genes and molecular pathways within neighbouring tumour-associated stroma in CRC cancer remain to date unexplored. A shift to the evaluation of the genetic and transcriptional phenotype of the environment surrounding cancer tissue is required to understand the complex molecular pathways underlying cancer initiation and metastatic progression.

Colonic epithelial cells lie in close proximity to the mesentery, primitive envelope of the colon, constituted by adipose tissue, nerves, blood vessels, and lymphatic vessels [21]. Advanced stages of CRC are characterised by the infiltration of the mesentery. Visceral white adipose tissue depots, including mesentery, have recently been recognised as a “metastatic niche”, harbouring poorly understood pro-metastatic characteristics [22]. Despite compelling evidence highlighting its role in cancer progression and therapy resistance, little is known of how the ECM is regulated.

In this study we sought to profile cancer related genes in human peritumour mesenteric adipose tissue and in distal mesenteric adipose tissue in colon cancer patients, seeking to identify differences in gene expression profile. Determining an aberrant pathway in the cancer cell-free stromal compartment may lead to identification of a genetic signature of the metastatic niche, leading to new therapeutic targets and novel prognostic and predictive biomarkers.

## 2. Results

RNA was extracted from 9 paired samples of peritumour adipose tissue (pAT) and distal adipose tissue. Out of the 9 paired samples, 6 paired samples were employed for Nanostring sequencing. Out of 6 patients histology of tumour tissue revealed one patient with colon cancer T2N0Mx, 4 patients with colon cancer pT3N0Mx, one patient with colon cancer pT4bN1Mx.

### 2.1. Nanostring Analysis

#### Clustering

In total, five samples (1, 2, 4, 6, 12) had lower RNA content than the others. In order to check that different RNA content had not introduced bias into the data hierarchical clustering and Principal Component Analysis (PCA) were observed (Supplementary Figures S1 and S2).

**Figure 1:**
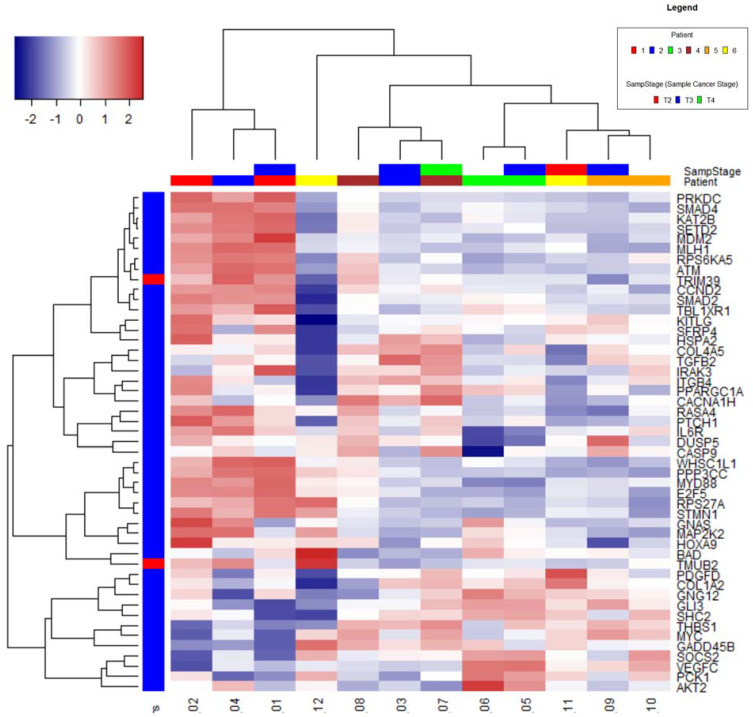
Hierarchical clustering for the 50 genes with highest variance. The heat map diagram shows the result of the two-way hierarchical clustering of genes and samples. Each row represents one gene and each column represents one sample. The gene clustering tree is shown on the left. The colour scale shown at the top-left illustrates the relative expression level of genes across all samples: blue colour represents an expression level below mean, red colour represents expression higher than the mean.

A heat map diagram was obtained by the hierarchical clustering for the 50 genes with highest variance performed on all samples (Figure 1).

Samples from the same patient are generally seen to cluster together (5 and 6, 9 and 10) or close to each other (1 and 2, 7, and 8). Samples 1, 2, 4, and 12 had the lowest RNA content and are separate from the other samples, indicating RNA content could be influencing the apparent expression of these genes.

Although samples 1, 2, and 4 cluster together in the PCA and hierarchical clustering, the other 2 samples (6 and 12) with low RNA content appear different, indicating that low RNA may not influence gene expression.

Missing data were within expected quality control (QC) range for all samples, all samples had > 94% field of view’s (FOV’s) counted (recommended minimum is 75% FOV’s). Binding density QC metrics were all within the range 0.25 to 0.39 (within recommended values of 0.1–1.8). The relationship between mean expression and standard deviation showed an approximately linear relationship at count levels well above the background. Control and housekeeping genes each had low standard deviation.

### 2.2. Cancer-Related Gene Expression in Peritumour Adipose Tissue and Distal Adipose Tissue

**Figure 2:**
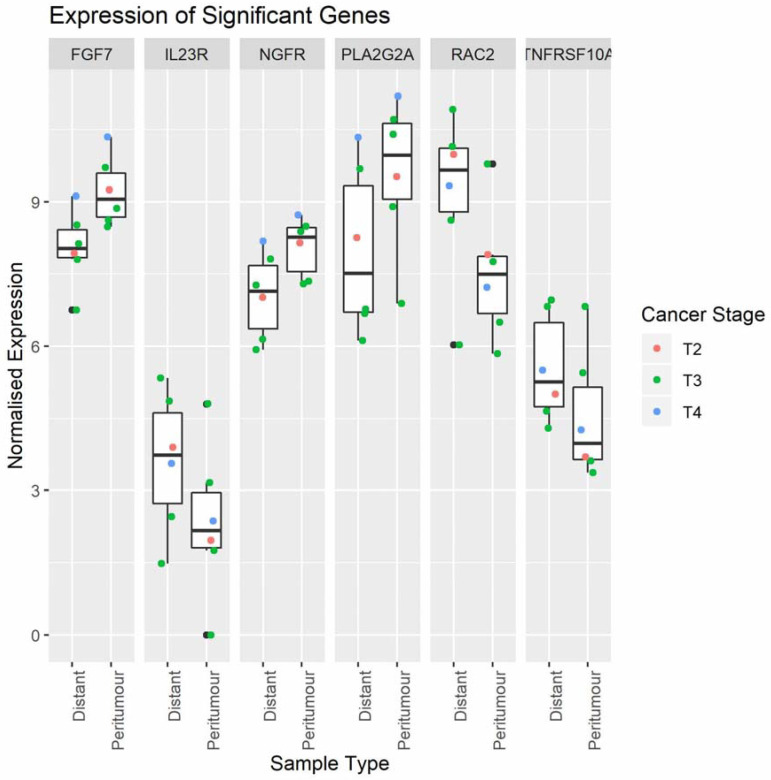
Boxplots showing normalised expression values for the six most significantly differentially expressed genes.

A total of 64 differentially expressed genes (DEGs) were identified in pAT compared to distal adipose tissue (Supplementary Table S1). The 6 most significantly DEGs were fibroblast growth factor 7 (*FGF7*), interleukin-23 (*IL23*), nerve growth factor receptor (*NGFR*), phospholipase A2, group IIA (*PLA2G2A*), ras-related C3 botulinum toxin substrate 2 (RAC2), tumour necrosis factor receptor superfamily member 10a (TNFRSF10A), (Figure 2). FGF7, NGFR, *PLA2G2A* were up-regulated, whilst *IL23* and *TNFRSF10A*, involved in inflammatory pathways, and *RAC2* were down-regulated.

A heatmap of DEGs is represented in Supplementary Figure S3.

### 2.3. Volcano Plot

**Figure 3:**
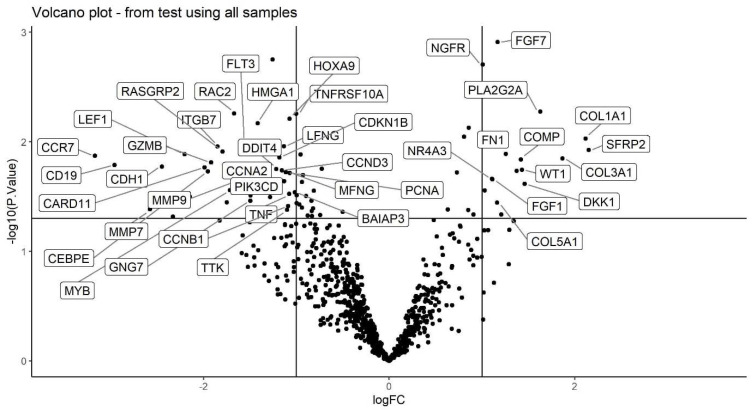
Volcano plot showing the log2 fold changes (Peritumour/Distant, x-axis) and −log10 *p*-values for all genes.

Log2 fold changes and –log10 *p*-values of all genes were plotted on x and y axes, respectively, to obtain the volcano plot in Figure 3. Represented genes have *p*-value < 0.05 and absolute log fold change greater than 1.

### 2.4. RT-PCR Validation of Differentially Expressed Genes Identified with PanCancer Panel

**Figure 4:**
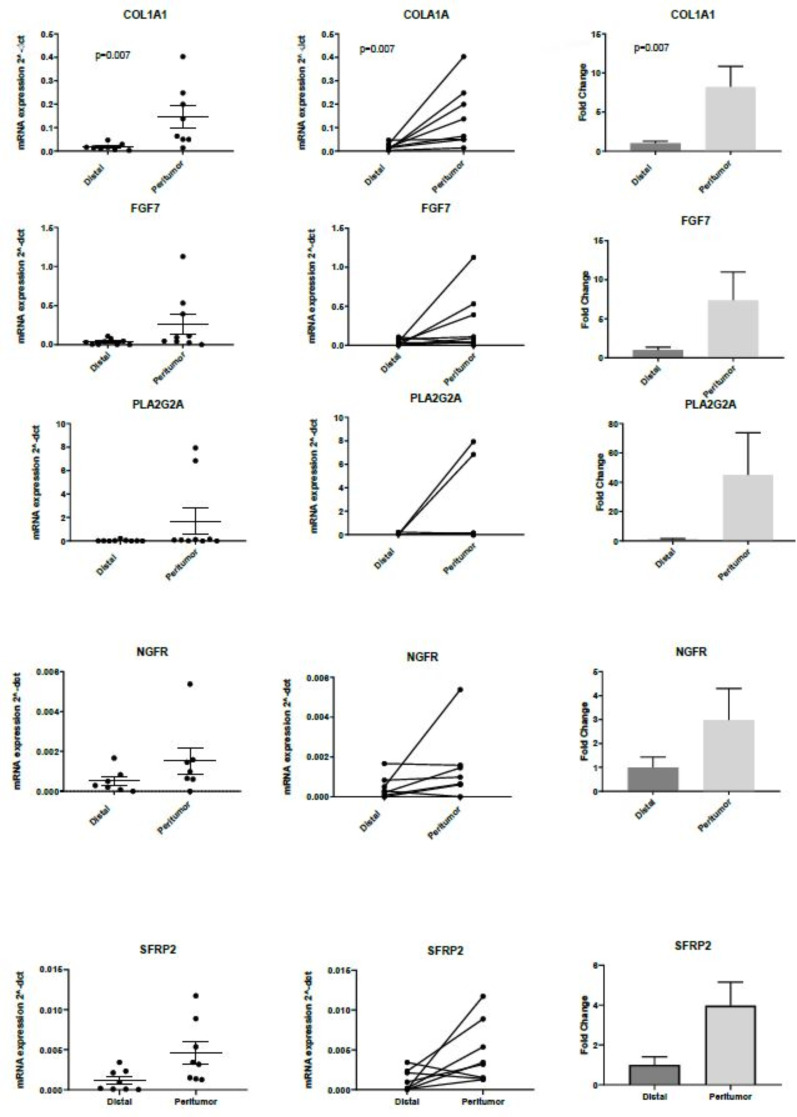
Validation of the 5 most significantly up-regulated genes identified with PanCancer pathway analysis. RT-PCR confirmed higher RNA expression of *COL1A1*, *FGF7* and *SFRP2* in pAT compared to distal AT (***COL1A1 n* = 8, *p* = 0.007; *FGF7 n* = 9, *p* = 0.05;** *PLA2G2A n* = 9, *p* = 0.16; *NGFR n* = 7, *p* = 0.10; ***SFRP2 n* = 8, *p* = 0.05**).

We identified 64 DEGs in pAT compared to distal adipose tissue employing the Nanostring nCounter PanCancer Pathway Panel. In total, five up-regulated genes with the highest logarithmic fold change were selected for validation with RT-PCR to include *COL1A1*, *FGF7*, *PLA2G2A*, *NGFR* and *SFRP2*. RT-PCR confirmed significant up-regulation of *COL1A1* (*p* = 0.007), *FGF7* (*p* = 0.05), and *SFRP2* (*p* = 0.054). Results are shown in Figure 4.

**Figure 5:**
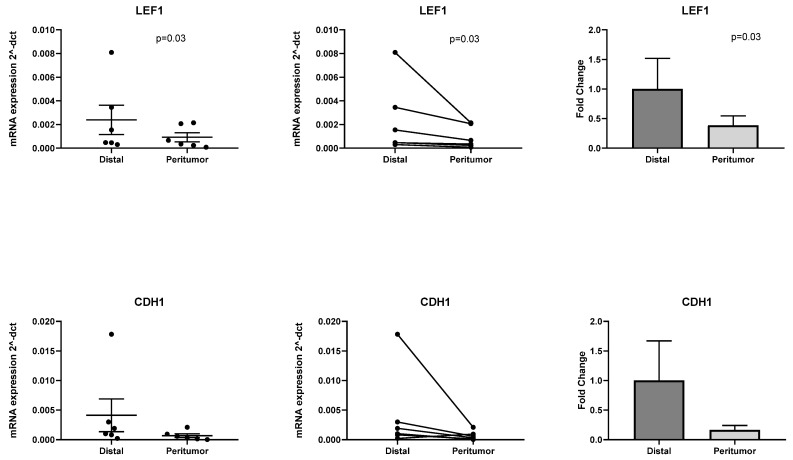
Validation with RT-PCR of 2 down-regulated genes identified with PanCancer pathway analysis (*LEF1 n* = 6, *p* = 0.03; *CDH1 n* = 6, *p* = 0.09).

*COL1A1* and *FGF7* are known profibrotic genes, whilst *SFRP2* is a known Wnt antagonist. Amongst the down-regulated genes we identified 2 genes involved in the Wnt pathway, *LEF1* and *CDH1*. Therefore, we further selected these 2 genes for validation with RT-PCR. We confirmed significant down-regulation in pAT of *LEF1* (*p* = 0.03) and down regulation of *CDH1*, although the *p* value was not significant (*p* = 0.09). Results are shown in Figure 5.

We observed aberrant transcription of genes related to the Wnt pathway (*SFRP2*, *LEF1,* and *CDH1*), indicating Wnt pathway downregulation. We also observed a fibrotic phenotype, characterised by up regulation of profibrotic genes, such as *COL1A1, FGF7,* and *SFRP2*.

We performed Kyoto Encyclopedia of Genes and Genomes (KEGG) enrichment analysis using ShinyGo for pathway identification. Up regulated genes in the pAT, identified amongst the 770 cancer-related genes included in the nCounter PanCancer Panel, were involved in the phosphatidylinositol-3-kinase (PI3K)-Akt signalling, focal adhesion, ECM-receptor interaction, mitogen-activated protein kinase (MAPK) signalling, Ras signalling, Wnt signalling, and Hippo signalling pathway (Supplementary Table S2).

Down regulated genes were involved in transcriptional regulation, cell cycle, PI3K-Akt signalling pathway, Notch signalling pathway, Wnt signalling pathway and mismatch repair (Supplementary Table S3).

### 2.5. Differential Immunohistochemical Expression of COL1A1 in Colon Cancer Tissue, Peritumour Mesenteric Adipose Tissue and Distal Adipose Tissue

We investigated protein expression of COL1A1 by immunohistochemistry (IHC) staining of colon cancer tissue, pAT and distal adipose tissue in 5 colon cancer patients. The IHC staining of COL1A1 was strongest in colon cancer tissue with thick bundles of collagen, becoming less intense in pAT, and weaker at distant adipose tissue. Interestingly, we observed a thicker collagen network in pAT compared to distal adipose tissue.

We focused on the collagen spatial distribution and architecture of adipose tissue. We observed thick collagen bundles in tumour slides, an organised collagen network surrounding adipose tissue lobes in pAT slides and a fine reticular appearance of collagen in distal adipose tissue. Based on such observation we elaborated a “collagen architectural score” reported in Methods and Materials section.

**Figure 6:**
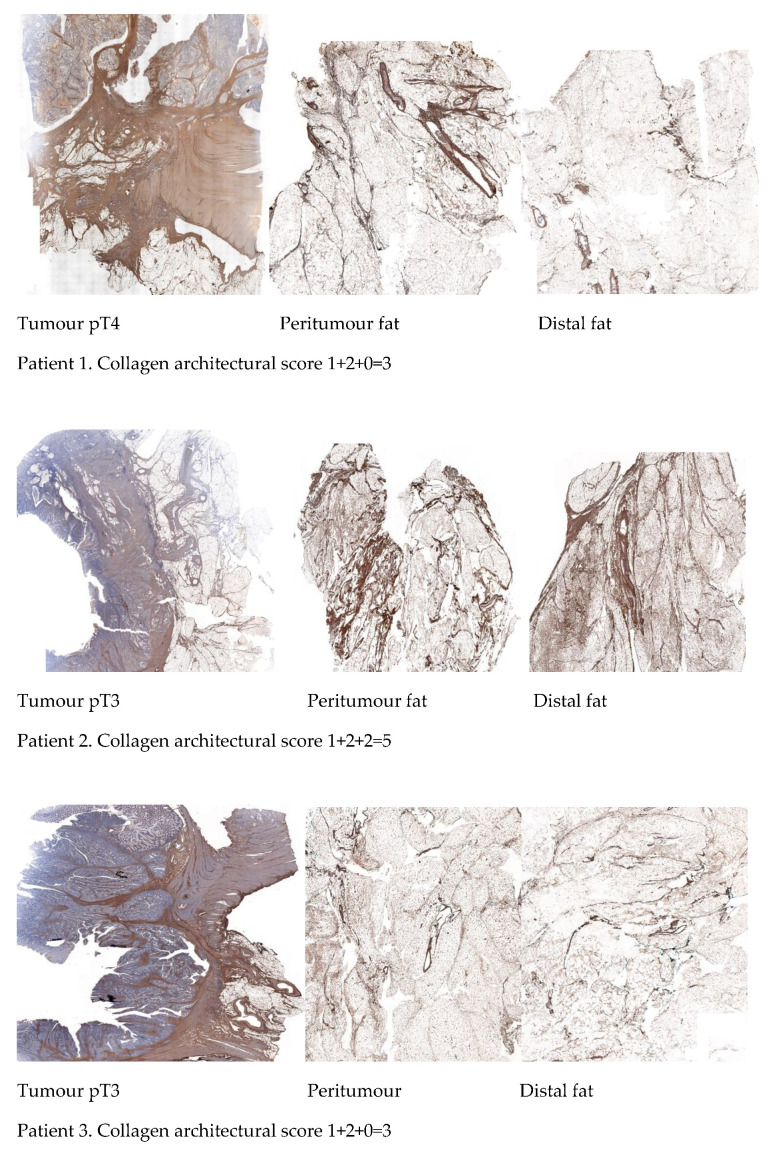

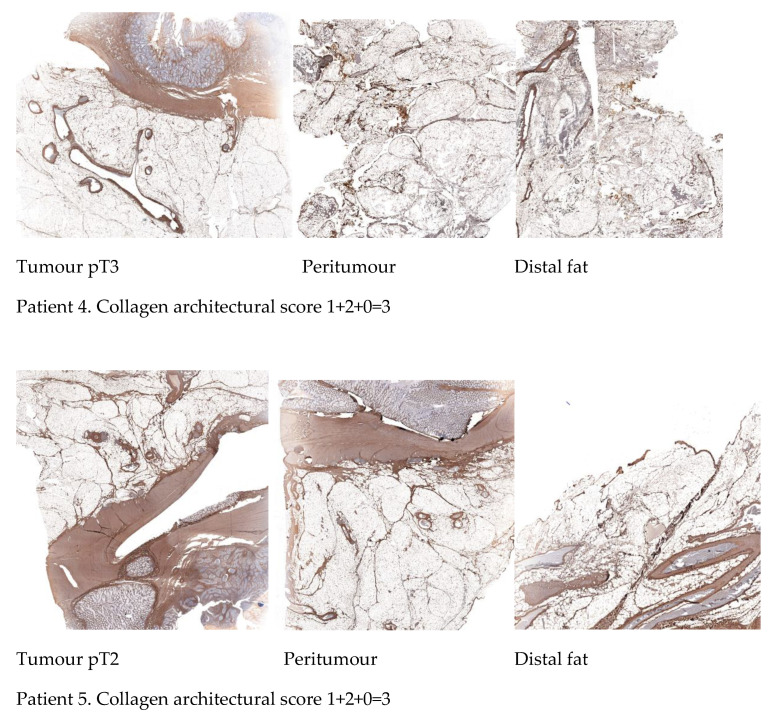
Immunohistochemical staining for COL1A1 expression in colon cancer tissue, peritumour adipose tissue and distal adipose tissue in 5 colon cancer patients. A “mesenteric collagen architectural score” based on collagen bundles and architecture of adipose tissue lobes is reported for each patient. We hypothesize COL1A1 up-regulation is mediated by down regulation of Wingless/Integrated (Wnt) pathway, contributing to the creation of a propagating mesenteric collagen network to reach target organs and facilitate engraftment and colonisation.

Images of IHC staining for COL1A1 expression in colon cancer tissue, pAT and distal adipose tissue, and “collagen architectural score” in 5 colon cancer patients are shown in Figure 6.

## 3. Discussion

Tumours are characterised by remodelling of the ECM, known as desmoplasia, with increased inflammatory cell infiltration and fibrosis contributing to tumour progression and metastasis [9]. In this exploratory study we sought to shift our attention from cancer cell related aberrant signalling pathways, to neighbouring cancer cell free mesenteric adipose tissue. For the first time, our results have highlighted down-regulation of the Wnt signalling pathway in peritumour cancer cell-free mesenteric adipose tissue compared to distal adipose tissue. We identified aberrant transcription of genes related to the Wnt pathway (*SFRP2*, *LEF1,* and *CDH1*). We observed up regulation of *SFRP2*, a recognised inhibitor of Wnt signalling [23] and down regulation of *LEF1*, a transcriptional activator of Wnt target genes. We also observed a fibrotic phenotype, characterised by up regulation of profibrotic genes, such as *COL1A1*, *FGF7,* and *SFRP2*. *FGF7* has been reported to be upregulated in CRC and mucosal field adjacent colorectal cancer. Interestingly, *FGFR2*, its downstream receptor, was reported down regulated in colorectal cancer tissue compared to controls, but not in distant sites, suggesting a role of *FGF7* within the peritumour microenvironment.

Genes involved in inflammatory pathways, including *IL23* and *TNFRS10A*, were down regulated in mesenteric pAT, suggesting that when cancer has invaded the mesentery fibrotic genes, rather than inflammatory genes, may be responsible of cancer progression and metastasis through a mesenteric route.

In this study, enrichment pathway analysis has highlighted that cancer cell-free mesenteric pAT is characterised by up regulation of pathways involved in ECM-receptor interaction and focal adhesion, whilst pathways involved in the cell cycle are down-regulated.

*COL1A1* was one of the most upregulated genes identified. Collagen is a major component of the ECM [24]. Collagen properties (fibre thickness, straight collagen bundles, and mechanical characteristics) have been demonstrated to influence cancer migration [25]. Several collagens, including collagen I, II, III, V, and IX, have been associated with tumour evolution and aggressive phenotypes in breast cancer [26,27]. Collagen-rich stroma has been associated with tumour cell invasion and metastasis in colon cancer [28,29]. Clinical studies in colon cancer patients have suggested that tumours with a greater stromal component have a poorer prognosis [30,31,32,33], attributed to secretion of cytokines, growth factors and angiogenic factors from stromal cells. The organisation of collagen network has been demonstrated to predict metastases [34]. Liang Y et al. reported that collagen arrangements with strong changes in collagen fibres affects the metastatic potential of CRC [35]. In this study we have identified a collagen network within the mesenteric pAT. We have identified, through IHC, a gradient of expression of COL1A1 gradually decreasing from cancer tissue to distant mesenteric pAT and a gradual modification in COL1A1 spatial arrangement. We have elaborated a “collagen architectural score” which could give clues to the metastatic potential. A higher score may indicate a more aggressive phenotype and may have a prognostic value. This scoring system needs to be validated with a larger sample size.

The tumour-stroma ratio has recently been demonstrated as a prognostic biomarker in colon cancer and has been suggested as an additional biomarker to the currently available and non-precise tumour, node, metastasis (TNM)staging system [36]. Collagen architecture within the mesentery may also represent a valid additional biomarker to the current TNM staging system.

Zhang Z et al. have demonstrated that *COL1A1* enhances the migratory potential of CRC cells through up-regulation of key genes involved in the Wnt/planar cell polarity (PCP) pathway involved in cytoskeletal rearrangements [37]. In our study we have observed up regulation of *COL1A1* and down regulation of the canonical Wnt/β-catenin pathway, raising the hypothesis of a possible cross talk between collagens and the canonical Wnt pathway resulting in cancer cell progression to distant organs.

All cell types in the colon originate from a single multipotent stem cell located at the base of the colonic crypt, regulated by Wnt and Notch signalling, involved in stem cell self-renewal, proliferation, and differentiation [38]. Aberrant canonical Wnt/β-catenin signalling pathway is one of the frequently identified alterations in CRC [39]. Wnt/β-catenin signalling is activated via genetic mutations of the adenomatous polyposis coli (*APC*) gene [40] or epigenetic changes, such as hypermethylation [41]. Non-canonical Wnt signalling is mediated through the PCP and the Wnt/calcium pathways. Non-canonical Wnt/calcium pathway may lead to cytoskeletal rearrangement [42].

In our study, for the first time, we report down regulation of Wnt pathway in pAT.

**Figure 7:**
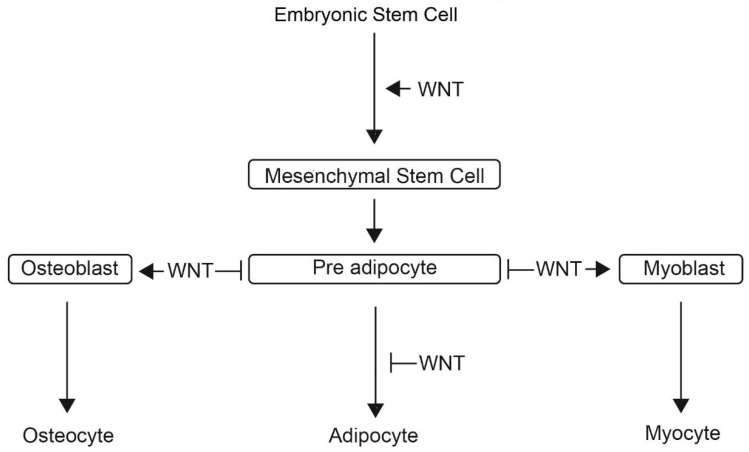
Schematic representation of the role of Wingless/Integrated (Wnt) signalling in embryonic stem cell and pre adipocyte differentiation. Wnt signalling promotes differentiation of embryonic stem cell in mesenchymal stem cell. At the next stage, Wnt antagonists induce differentiation of pre adipocyte into mature adipocytes.

Wingless-type mouse mammary tumour virus (MMTV) integration site family members (Wnts) are a family of secreted glycoproteins involved in mesodermal development, induction of differentiation from embryonic stem cell to mesenchymal stem cell, regulation of tissue homeostasis, and remodelling [43,44,45]. The function of Wnt signalling in cell differentiation is depicted in Figure 7.

Adipose derived mesenchymal stem cells (MSC) have been demonstrated to increase metastatic properties of colon cancer cells [46]. Chan et al., have demonstrated that adipose-derived stem cells fuse with cancer cells generating cells with increased tumorigenic potential [47].

Interactions between cancer cell (seed) and organ microenvironment (soil) has led to the “seed and soil” hypothesis [48]. Kaplan et al., in 2005, demonstrated that specific vascular endothelial growth factor receptor 1(VEGFR1)+ cells form permissive niches in distant organs and following tumour implantation local fibroblasts induce production of fibronectin [49]. Instead, we speculate that Wnt down regulation is induced by migrating cancer cells and cancer stem cells (CSC) in order to maintain a pool of undifferentiated stem cells leading to migration and metastasis. We postulate that colon cancer cells detaching from primary tumours form a complex with MSCs and pre adipocytes, allowing migration to more distant sites through a mesenteric collagen network. This hypothesis is supported by a study demonstrating that metastatic cells can bring their own soil, consisting of stromal components including activated fibroblasts, from the primary site to the lungs [50]. It has been reported that *SFRP2* suppression of Wnt signalling mediates MSC self-renewal promoting cell engraftment and myocardial repair [51]. We speculate that Wnt downregulation in the pAT may be acquired for maintenance and self-renewal of MSC and preadipocytes committed to the creation of a fibrotic stromal environment with increased expression of ECM proteins, contributing to the creation of a pre-metastatic niche. We hypothesise that homing in target distant organ is favoured by migrating cancer cell, MSC and pre-adipocyte complex with local production of ECM proteins once they have reached target organs. Unfortunately, in our study we did not evaluate the cell type of origin of *SFRP2*. Nevertheless, we hypothesise that *SFRP2* overexpressing MSC and pre-adipocytes are involved in initiating a metastatic niche through up-regulation of collagen, contributing to the creation of a propagating mesenteric collagen network to reach target organs and facilitate engraftment and colonisation. Interestingly, type I collagen has also been demonstrated to increase engraftment of cancer cells in mice, possibly mediated by increase in stem cells induced by abundant collagen. Moreover, it has been reported that type 1 collagen inhibits differentiation and favours a stem-cell like phenotype in human CRC cells [52]. These studies, highlighting a link between collagen and stem cells, support the findings of our study. Results from our study have revealed both collagen type 1 upregulation and Wnt pathway down regulation, both responsible for a stem-cell phenotype. In our study we have identified up regulation of *SFRP2*, a mesenchymal and pre-adipocyte marker, and also a pro-fibrotic gene involved in collagen production. We have identified, through IHC, a gradient of expression of COL1A1, more abundant in cancer tissue compared to normal distant sites, supporting its role in migration. Up-regulation of collagen proteins has been reported in colorectal liver metastases compared with normal liver tissue, supporting our hypothesis that collagen may represent a vector for progression from primary tumour site to distant organs [53].

The metastatic process is a complex multistep process characterised by initial epithelial-mesenchymal-transition (EMT) favouring detachment of cancer cells from the ECM, followed by migration into the systemic circulation with final homing in distant organs, favoured by mesenchymal-epithelial transition (MET). Up regulation of Wnt/β-catenin pathway promotes EMT-associated differentiation, whilst inhibition of Wnt signalling has been reported to be associated with MET [54]. In our study, amongst EMT associated genes in pAT, *CDH1*, an epithelial marker, was down regulated compared to distal adipose tissue, whilst EMT inducers, such as *FGF7*, *FGF1*, *TGFβ2* were up regulated, suggesting a EMT phenotype in pAT. In our study *FGF1* and *TGFβ2* up-regulation, identified with Nanostring sequencing, were not evaluated with RT-PCR. Other known mesenchymal markers, such as zinc finger transcription factors Snail1 and Snail2, Zinc finger E-box binding homeobox (ZEB)1 and ZEB2, twist family basic helix-loop-helix (bHLH) transcription factor (TWIST)1 [55] were not included in the Nanostring assay. Results of this study may indicate that at more distant mesenteric sites transcriptional changes start occurring favouring reversal of EMT in favour of a MET phenotype.

Stem cells are highly plastic and dynamic cells, exhibiting the capability of shifting between quiescence and proliferation and EMT and MET [56]. The switch between CSC and non-CSC has also been reported in CRC [57]. Wnt activation is involved in CSC formation through de-differentiation of originating stem cell [58]. In the neighbouring mesenteric pAT, we postulate a switch from Wnt up-regulation to Wnt-down regulation to maintain a pool of stem cells necessary to create a highly dynamic microenvironment favourable to migration.

The microenvironment has the ability to determine fate and function of cancer cells. Our study has identified two potential mechanisms involved in cancer cell fate and function, including Wnt inactivation and desmoplasia mediated by *COL1A1*. This newly reprogrammed microenvironment is responsible for stem cell maintenance and represents the first step of metastasis with the creation of a stem cell niche. Why CRC cells preferentially metastasise to the liver and lung remains to be determined. We hypothesise that committed stem cells are involved in preferential target organ homing.

Patients with CRC remain at high risk of recurrent disease following surgery with curative intent [59]. Radical surgery in gastrointestinal cancers consists in en bloc removal of primary tumour with its lympho-vascular drainage by excision of organ-specific mesentery known as total mesorectal excision (TME) or complete mesocolic excision (CME) [60,61]. Post CRC surveillance is based on endoscopic follow up to detect metachronous pre-malignant polyps and lesions not previously detected [62]. However, the effectiveness of current surveillance strategies based on adenoma detection has not been ascertained and appropriate risk stratification for CRC survivors is needed [63]. 

The field cancerisation theory asserts that cancer arises from a genetically altered mucosa [18]. We would like to translate this concept to the mesentery and postulate a “mesenteric field metastatisation theory”, hypothesising that gene expression profile in mesenteric pAT and distal mesenteric adipose tissue may provide clues of the metastatic potential of colon cancer and may have a role in colon cancer staging and risk stratification. Gene expression profile of the mesentery at the resected margins may be the repository of metastatic properties and provide clues of aggressive phenotype. Aberrant gene expression at the mesenteric resected margin may help to identify colon cancer patients undergoing surgery with curative intent at high risk of local and distant recurrence of disease requiring early and intensive endoscopic and radiologic surveillance. Results from our study show a potential value of *SFRP2* and *COL1A1* to identify patients at high risk of colon cancer recurrence and metastatic disease. To confirm our hypothesis of *SFRP2* and *COL1A1* involvement in cancer cell and MSC complex migration and engraftment in distant organs, it would be necessary to confirm *COL1A1* and *SFRP2* expression in liver metastases from CRC.

*SFRP2* has key characteristics of matricellular proteins and has been suggested as a candidate therapeutic target in cancer [64]. A humanised *SFRP2* monoclonal antibody has been demonstrated to reduce angiosarcoma and breast cancer growth in mice models [65]. Certainly, the role of *SFRP2* in the context of colon cancer requires investigation and may represent a therapeutic target. A biological difference between colon cancer and rectal cancer is widely recognised [66], therefore in our study we have excluded rectal cancer patients. However, we suggest that the principle of our study also requires validation in rectal cancer patients.

We acknowledge the limited sample set of our study (*n* = 9). Larger studies are required to confirm Wnt pathway down-regulation in pAT and elucidate its role on cancer cell progression. In vitro studies are needed to identify the cell population expressing *SFRP2* within pAT. Moreover, the role of *SFRP2* in collagen synthesis requires further investigation. Mesenteric gene expression profile represents a promising candidate for both prognostic biomarkers for aggressive disease and for new therapeutic targets.

## 4. Materials and Methods

### 4.1. Patients

Samples were collected through Arden Tissue Bank Ethics. All patients enrolled provided written informed consent and were enrolled in a pre-existing clinical study ethically approved from Coventry and Warwick Local Research Ethics Committee (REC: 09/H1211/38, 26th August 2016). The study was performed in accordance to the Declaration of Helsinki.

Overall, nine patients with radiological evidence of colon cancer stage T3/T4 undergoing surgery were identified through theatre lists. Patients with rectal cancer were excluded from the study. All patients had histologically confirmed adenocarcinoma. Clinical-pathological characteristics of colon cancer patients are given in Table 1.

### 4.2. Tissue collection and RNA Extraction

Resected colonic specimens were collected by a Tissue Bank Assistant and transferred to Pathology Department immediately after resection. From each resected colonic specimen approximately 0.1 g of adipose tissue was resected from mesentery adjacent to the tumour and from distal mesentery at approximately 10 cm by one experienced Pathologist. Site of peritumour adipose tissue resection was marked with yellow ink. Colon cancers with disease involvement of the mesentery were excluded from the study. Adipose tissue was immediately placed in a 50 mL centrifuge tube containing Hank’s Balanced Salt Solution (HBSS). Adipose tissue was collected from the Pathology department and processed within one hour. The cancer-cell free adipose tissue was ascertained by Pathologists.

In 6 paired samples RNA was extracted using RNeasy lipid tissue kit (Qiagen, Hilden, Germany). Mesenteric adipose tissue was lysed in Qiazol Lysis Reagent (sample:lysis buffer 1:5) using a tissue homogeniser (Starlab, Milton Keynes, UK). 10 μL of β-mercaptoethanol was added for every 1 mL of lysis buffer. After addition of 200 μL of chloroform for every 1 mL of sample and vigorous shaking for 15 s the homogenate was centrifuged at 4 °C for 15 min at 12,000× *g* leading to separation into aqueous and organic phases. The upper aqueous phase (RNA) was carefully extracted and 1 volume 75–100% alcohol was added to provide optimal binding conditions.

The sample was applied to the RNeasy spin column. 350 μL of Buffer RW1 was added to the RNeasy column. 80 μL of DNAse solution (10 μL DNAse +70 μL Buffer RDD per sample) was added directly to column. After 15 min 350 μL of Buffer RW1 was added to the RNeasy column. 500 μL of RPE Buffer was added twice to the RNeasy column. Spin columns were dry centrifuged for 2 min at maximum speed 13,500× *g*. 15 μL of RNase-free water was added directly onto the membrane and centrifuged for 2 min. The liquid was reapplied onto the membrane and centrifuged for 5 min.

The DNAse-free RNA was quantified spectrophotometrically by measuring absorbance at 230, 260, and 280 nm (NANOdrop, Termo Fisher Scientific, Waltham, MA, USA). When the A260–280 ratio was between 1.8 and 2.2, the isolated RNA was qualified as pure and used in subsequent experiments. Samples were labelled and stored at −80 °C.

In 3 paired samples total RNA was extracted employing Trizol reagent, followed by a DNAse step. Trizol was added to sample (0.75 mL of Trizol per 0.25 mL of sample). 0.2 mL of chloroform per 0.75 mL of Trizol LS Reagent was added and incubated for 3 min. Sample was centrifuged for 15 min at 12,000× *g* at 4 °C. The upper aqueous phase containing RNA was transferred to a new tube. Then, 0.5 mL of isopropanol was added to the aqueous phase per 0.75 mL of Trizol LS reagent, incubated for 10 min and centrifuged for 30 min at 13,000× *g* at 4 °C. The pellet was resuspended in 1 mL of 75 % ethanol per 0.75 mL of Trizol. The sample was centrifuged for 30 min at 13,000× *g* at 4 °C. Supernatant was discarded and the RNA pellet was air dried for 10–15 min. The pellet was resuspended in 10 μL of RNase-free water and quantified spectrophotometrically by measuring absorbance at 230, 260, and 280 nm (NANOdrop).

cDNA was synthesised using reverse transcription. RNA (1 g) was mixed with 1 μL random hexamers (Thermo Fisher Scientific) and 1 μL dNTP mix (Promega, Madison, WI, USA) to a final volume of 12 μL by adding RNAse-free water. Samples were heated at 70 °C for 10 min before chilling on ice. Subsequently, 8μL of mixture containing RNase inhibitor, reverse transcriptase (RT) buffer (Sigma-Aldrich, St. Louise, MO, USA), reverse transcriptase (Sigma-Aldrich) and RNase-free water were added to each sample. Samples were heated at 25 °C for 10 min, 37 °C for 50 min and 80 °C for 10 min. 180 μL of RNAse free water were added to the cDNA to a final volume of 200 μL and stored at −20 °C.

Quantitative Real-Time PCR assays were performed using Sybr Green JumpStart (Sigma–Aldrich). Transcript abundance was measured with an Applied Biosystems 7500 RealTime PCR system (Applied Biosystem, Foster City, CA, USA). The variances of input cDNA were normalised against housekeeping gene L19. Melting curve analysis confirmed amplification specificity. Primer sequences (Thermo Fisher Scientific) are reported in Supplementary Table S4.

For data analysis, a ∆C*t* was calculated based on the difference between L19 and the target gene. Gene expression was calculated based on the following formula:

mRNA expression = 2^−∆C*t*^, where ∆C*t* = target gene C*t* − L19 Ct

### 4.3. Transcriptomic Analyses—Nanostring nCounter Pre-Designed Gene Expression Panel

To explore the differences in cancer-related gene profile in pAT and distal adipose tissue we employed the Nanostring nCounter PanCancer Pathway Panel, evaluating RNA expression levels of 770 essential genes. Out of the 9 paired samples, 6 paired samples were employed for Nanostring sequencing. The nCounter Analysis System is based on a digital color-coded barcode technology which allows for direct multiplexed measurement of gene expression from low amount of mRNA (25 to 300 ng) without need for amplification. Data import, Quality Control (QC) and normalisation were carried out in R with the assistance of the R packages NanoStringNorm and Limma.

The data deposition is in progress in Gene Expression Omnibus.

### 4.4. Normalisation

A comprehensive check of normalisation methods applicable to the raw data was carried out using the norm.comp functionality of the NanoStringNorm R package. From this analysis, the most appropriate normalisation was selected based on intraclass correlation coefficient (ICC) and *z*-scores. The normalisation chosen used the following criteria:

- CodeCount = sum. Correction for global differences between samples.
- Background = mean. How the negative control probes are used for background correction.
- SampleContent = top.geo.mean.
- OtherNorm = none

The normalisation performed well, reducing the gene coefficient of variation (Supplementary Figure S4) and reducing global signal differences between samples (Supplementary Figure S5).

### 4.5. Statistical Analysis

To look for differences in gene expression between peritumour and distant adipose tissue a paired t-test for differential expression was applied using the R package Limma. Statistical analysis of RT-PCR data was performed using GraphPad Prism^®^. Expression levels were normalised to L19. Significant differences in transcript abundance between peritumour and distal adipose tissue were tested using the non-parametric Wilcoxon signed ranks test with cut-off of *p*-value < 0.05.

### 4.6. Immunohistochemistry

Colon cancer, pAT, and distal adipose tissue sections were incubated with anti-COL1A1 antibody (ab34710, Abcam, Cambridge, UK) in a dilution of 1:200. Sections were developed using Novolynk Polymer Detection System (Leica, Wetzlar, Germany). To demonstrate specific binding, the primary antibody was omitted for negative control.

We have elaborated a “collagen architectural score” based on collagen distribution and architecture of adipose tissue lobes observed at tumour site, at pAT site and at distal adipose tissue site.

A value of 1 was assigned for the presence of each parameter (thick collagen bundles and organised adipose tissue lobes) and 0 for the absence at each location (tumour, pAT, and distal adipose tissue). The total score is derived through arithmetical sum of single parameters for each location (Table 2).

## 5. Conclusions

The present study provides the first gene expression profile of cancer associated genes in human colon cancer pAT. Results have shown aberrant transcription of genes in cancer free pAT most likely contributing to cancer progression rather than cancer cell proliferation. We have observed down regulation of Wnt pathway related genes, a well-known upregulated pathway in CRC. Furthermore, we have identified a fibrotic phenotype of the cancer free pAT characterised by up-regulation of *COL1A1*. Additionally, we have shown up regulation of *SFRP2*, a mesenchymal and pre-adipocyte marker, and also a pro-fibrotic gene involved in collagen production, exhibiting pro-collagenase enhancing activity of Tolloid-like metalloproteinases [67].

Identifying key molecules and signalling pathways within the stromal compartment has great potential in colon cancer with metastatic phenotype. The value of the results of this exploratory study is related to the identification of novel stromal gene signatures that could represent critical therapeutic targets and which could help identify high risk patients for metastatic spread.

## Figures and Tables

**Table 1:** Demographic characteristics of the cohort of patients with colon cancer (*n* = 9).

Characteristic	Total Colon Cancer Patients (*n* = 9)
Age at diagnosis (years), mean ± SD	71.22 ± 9.44
Gender	
Male, *n* (%)	6 (66.6)
Female, *n* (%)	3 (33.3)
Cancer location	
Caecum, *n* (%)	1(11.11)
Transverse colon, *n* (%)	3 (33.3)
Sigmoid colon, *n* (%)	5 (55.5)
Tumour type	
Adenocarcinoma well and moderately differentiated	1 (11.11)
Adenocarcinoma moderately differentiated	7 (77.77)
Adenocarcinoma poorly differentiated	1 (11.11)
Depth of invasion (pT)	
pT2	2 (22.2)
pT3	6 (66.6)
pT4	1 (11.1)
Nodal involvement (pN)	
N0	7 (77.7)
N1	2 (22.2)
Systemic metastasis (M)	
M0	8
M1a	1
TNM stage at diagnosis	
Stage I	2 (22.2)
Stage II	4 (44.44)
Stage III	2 (22.22)
Stage IV	1 (11.11)

SD: standard deviation; pT: pathological Tumour stage pN: pathological nodes; M: metastasis; TNM: tumour, node, metastasis.

**Table 2:** Mesenteric collagen architectural score in colon cancer.

Parameters	Sub-Score *
Collagen spatial distribution	0 = absence of thick collagen bundles
	1 = presence of thick collagen bundles
Organisation of adipose tissue lobes	0 = absence of organised adipose tissue lobes
	1 = presence of organised adipose tissue lobes

* The total score is derived through arithmetical sum of single parameters at tumour, peritumour adipose tissue and distal adipose tissue site.

## Data Availability

The data presented in this study is available in supplementary material. The data deposition is in progress in Gene Expression Omnibus.

## Supplementary Materials

The following are available online at https://www.mdpi.com/1422-0067/22/5/2430/s1. Figure S2. Principal component analysis plot showing PCs 1 and 2 for all samples, Figure S4. Change in the distribution of gene coefficient of variation on normalisation of data, Figure 5. Density plots for all samples of raw (left) and normalised (right) data, Table S1. List of 64 differentially expressed genes between peritumour adipose tissue and distal adipose tissue identified within 770 cancer related genes, Table 2. The table shows KEGG pathways associated with up-regulated genes obtained with ShinyGo, Table S3. The table shows KEGG pathways associated with down-regulated genes obtained with ShinyGo, Table S4. Forward and reverse primers.
