# Supplementary material for: Fibrotic Phenotype of Peritumour Mesenteric Adipose Tissue in Human Colon Cancer: A Potential Hallmark of Metastatic Properties
Source: Int J Mol Sci. 2021 Feb 28;22(5):2430. doi: 10.3390/ijms22052430 (PMC7957668; doi:10.3390/ijms22052430)
Supplement: Supplementary file 1 [file ijms-22-02430-s001.zip › Supplementary Figure 2.docx]

Supplementary Figure 2. Principal component analysis plot showing PCs 1 and 2 for all samples.
