# Supplementary material for: Fibrotic Phenotype of Peritumour Mesenteric Adipose Tissue in Human Colon Cancer: A Potential Hallmark of Metastatic Properties
Source: Int J Mol Sci. 2021 Feb 28;22(5):2430. doi: 10.3390/ijms22052430 (PMC7957668; doi:10.3390/ijms22052430)
Supplement: Supplementary file 1 [file ijms-22-02430-s001.zip › Supplementary Table 1.docx]

Supplementary Table 1. List of 64 differentially expressed genes between peritumour adipose tissue and distal adipose tissue identified within 770 cancer related genes.

Gene name Accession logFC AveExpr t p-value adj.p v

| FGF7 | NM_002009.3 | 1.167 | 8.626 | 5.064 | 0.001 | 0.406 |
| --- | --- | --- | --- | --- | --- | --- |
| NGFR | NM_002507.1 | 1.008 | 7.560 | 4.669 | 0.001 | 0.406 |
| PLA2G2A | NM_000300.2 | 1.628 | 8.787 | 3.900 | 0.005 | 0.406 |
| RAC2 | NM_002872.3 | -1.670 | 8.334 | -3.868 | 0.005 | 0.406 |
| TNFRSF10A | NM_003844.2 | -1.002 | 5.036 | -3.866 | 0.005 | 0.406 |
| HOXA9 | NM_152739.3 | -1.074 | 5.091 | -3.785 | 0.006 | 0.406 |
| HMGA1 | NM_145904.1 | -1.415 | 6.312 | -3.717 | 0.006 | 0.406 |
| COL4A5 | NM_033381.1 | 0.858 | 6.311 | 3.648 | 0.007 | 0.406 |
| IL1R1 | NM_000877.2 | 0.807 | 8.646 | 3.510 | 0.009 | 0.406 |
| COL1A1 | NM_000088.3 | 2.116 | 8.391 | 3.485 | 0.009 | 0.406 |
| FLT3 | NM_004119.1 | -1.563 | 3.88 | -3.440 | 0.009 | 0.406 |
| LFNG | NM_001040168.1 | -1.135 | 5.238 | -3.369 | 0.010 | 0.406 |
| ITGB7 | NM_000889.1 | -1.851 | 5.527 | -3.366 | 0.011 | 0.406 |
| SFRP2 | NM_003013.2 | 2.150 | 9.897 | 3.314 | 0.011 | 0.406 |
| RASGRP2 | NM_001098670.1 | -1.796 | 7.7980 | -3.290 | 0.012 | 0.406 |
| FN1 | NM_212482.1 | 1.257 | 9.910 | 3.255 | 0.012 | 0.406 |
| LEF1 | NM_016269.3 | -2.205 | 6.147 | -3.253 | 0.012 | 0.406 |
| RASA4 | NM_001079877.2 | -0.955 | 7.917 | -3.250 | 0.013 | 0.406 |
| CCR7 | NM_001838.2 | -3.172 | 5.334 | -3.229 | 0.013 | 0.406 |
| CDKN1B | NM_004064.2 | -1.183 | 5.720 | -3.207 | 0.013 | 0.406 |
| COL3A1 | NM_000090.3 | 1.867 | 11.904 | 3.189 | 0.014 | 0.406 |
| COMP | NM_000095.2 | 1.418 | 4.072 | 3.173 | 0.014 | 0.406 |
| GZMB | NM_004131.3 | -1.920 | 6.832 | -3.130 | 0.015 | 0.406 |
| CD19 | NM_001770.4 | -2.961 | 6.121 | -3.094 | 0.016 | 0.406 |
| CDH1 | NM_004360.2 | -2.450 | 3.699 | -3.071 | 0.016 | 0.406 |
| CARD11 | NM_032415.2 | -1.991 | 3.775 | -3.059 | 0.017 | 0.406 |
| MAP2K6 | NM_002758.3 | -0.727 | 6.743 | -3.039 | 0.017 | 0.406 |
| DDIT4 | NM_019058.2 | -1.217 | 8.539 | -3.036 | 0.017 | 0.406 |
| WT1 | NM_000378.3 | 1.431 | 5.017 | 3.025 | 0.017 | 0.406 |
| CCND3 | NM_001760.2 | -1.155 | 8.339 | -3.018 | 0.018 | 0.406 |
| CEBPE | NM_001805.2 | -1.952 | 3.189 | -3.001 | 0.018 | 0.406 |
| PCNA | NM_002592.2 | -1.114 | 5.825 | -2.989 | 0.018 | 0.406 |
| THBS1 | NM_003246.2 | 0.728 | 11.874 | 2.985 | 0.019 | 0.406 |
| MFNG | NM_002405.2 | -1.072 | 6.841 | -2.980 | 0.019 | 0.406 |
| CDKN2D | NM_001800.3 | -0.929 | 5.760 | -2.950 | 0.020 | 0.414 |
| NR4A3 | NM_173198.1 | 1.108 | 8.504 | 2.897 | 0.021 | 0.414 |
| FGF1 | NM_033137.1 | 1.112 | 5.856 | 2.888 | 0.0219 | 0.414 |
| CCNA2 | NM_001237.2 | -1.313 | 5.460 | -2.866 | 0.022 | 0.414 |
| MMP9 | NM_004994.2 | -1.642 | 8.168 | -2.861 | 0.022 | 0.414 |
| PIK3CD | NM_005026.3 | -1.132 | 7.345 | -2.860 | 0.022 | 0.414 |
| DKK1 | NM_012242.2 | 1.459 | 4.337 | 2.823 | 0.024 | 0.421 |
| MYB | NM_005375.2 | -1.716 | 4.846 | -2.735 | 0.027 | 0.446 |
| DLL1 | NM_005618.3 | -0.810 | 6.431 | -2.727 | 0.027 | 0.446 |
| BAIAP3 | NM_003933.4 | -1.016 | 4.141 | -2.704 | 0.028 | 0.447 |
| TNF | NM_000594.2 | -1.077 | 5.038 | -2.681 | 0.029 | 0.447 |
| HOXA10 | NM_018951.3 | -0.996 | 5.844 | -2.661 | 0.030 | 0.447 |
| H2AFX | NM_002105.2 | -0.895 | 5.219 | -2.650 | 0.031 | 0.447 |
| GNG7 | NM_052847.1 | -1.495 | 4.477 | -2.586 | 0.034 | 0.465 |
| SIRT4 | NM_012240.1 | -0.836 | 3.312 | -2.585 | 0.034 | 0.465 |
| COL5A1 | NM_000093.3 | 1.163 | 9.217 | 2.557 | 0.035 | 0.465 |
| POLD4 | NM_021173.2 | -0.998 | 8.893 | -2.552 | 0.036 | 0.465 |
| EZH2 | NM_004456.3 | -0.968 | 5.939 | -2.534 | 0.037 | 0.471 |
| CCNB1 | NM_031966.2 | -1.087 | 5.939 | -2.509 | 0.038 | 0.475 |
| FANCE | NM_021922.2 | -0.939 | 4.524 | -2.487 | 0.039 | 0.475 |
| GPATCH3 | NM_022078.2 | -0.819 | 4.683 | -2.476 | 0.040 | 0.475 |
| MMP7 | NM_002423.3 | -2.578 | 3.785 | -2.465 | 0.041 | 0.475 |
| TGFB2 | NM_003238.2 | 0.631 | 5.012 | 2.461 | 0.041 | 0.475 |
| TTK | NM_003318.3 | -1.098 | 3.755 | -2.458 | 0.041 | 0.475 |
| RET | NM_020630.4 | 0.852 | 3.536 | 2.457 | 0.041 | 0.475 |
| RASGRF2 | NM_006909.1 | -0.834 | 5.000 | -2.442 | 0.042 | 0.479 |
| GLI1 | NM_005269.1 | -0.502 | 3.758 | -2.428 | 0.0436 | 0.4819 |
| DTX1 | NM_004416.2 | -0.743 | 6.596 | -2.386 | 0.046 | 0.491 |
| PRDM1 | NM_182907.1 | -0.876 | 7.973 | -2.377 | 0.047 | 0.491 |
| SMARCB1 | NM_003073.3 | -0.831 | 8.187 | -2.370 | 0.047 | 0.491 |
