# Supplementary material for: Fibrotic Phenotype of Peritumour Mesenteric Adipose Tissue in Human Colon Cancer: A Potential Hallmark of Metastatic Properties
Source: Int J Mol Sci. 2021 Feb 28;22(5):2430. doi: 10.3390/ijms22052430 (PMC7957668; doi:10.3390/ijms22052430)
Supplement: Supplementary file 1 [file ijms-22-02430-s001.zip › Supplementary Table 2.docx]

Supplementary Table 2. The table shows KEGG pathways associated with up-regulated genes obtained with ShinyGo.

| **Enrichment FDR** | **Genes in list** | **Total genes** | **Functional Category** |
| --- | --- | --- | --- |
| 1.8E-09 | 8 | 353 | PI3K-Akt signaling pathway (COL1A1 COL4A1 COMP FGF1 FGF7 FN1 NGFR THBS1) |
| 1.8E-09 | 6 | 96 | Amoebiasis (COL1A1 COL3A1 COL4A1 FN1 IL1R1 TGFB2) |
| 4.4E-08 | 5 | 82 | ECM-receptor interaction (COL1A1 COL4A1 COMP FN1 THBS1) |
| 8.6E-08 | 5 | 99 | AGE-RAGE signaling pathway in diabetic complications (COL1A1 COL3A1 COL4A1 FN1 TGFB2) |
| 2.3E-06 | 5 | 199 | Focal adhesion (COL1A1 COL4A1 COMP FN1 THBS1) |
| 3.3E-06 | 4 | 90 | Protein digestion and absorption (COL1A1 COL3A1 COL4A1 COL5A1) |
| 9.0E-06 | 6 | 523 | Pathways in cancer (COL4A1 FGF1 FGF7 FN1 RET TGFB2) |
| 9.8E-06 | 5 | 295 | MAPK signaling pathway (FGF1 FGF7 IL1R1 NGFR TGFB2) |
| 1.3E-05 | 5 | 318 | Human papillomavirus infection (COL1A1 COL4A1 COMP FN1 THBS1) |
| 2.1E-05 | 3 | 48 | Malaria (COMP TGFB2 THBS1) |
| 4.8E-05 | 4 | 205 | Rap1 signaling pathway (FGF1 FGF7 NGFR THBS1) |
| 6.9E-05 | 4 | 230 | Ras signaling pathway (FGF1 FGF7 NGFR PLA2G2A) |
| 3.2E-04 | 3 | 130 | Relaxin signaling pathway (COL1A1 COL3A1 COL4A1) |
| 4.3E-04 | 3 | 148 | Gastric cancer (FGF1 FGF7 TGFB2) |
| 7.9E-04 | 3 | 186 | Transcriptional misregulation in cancer (NGFR WT1 NR4A3) |
| 8.8E-04 | 3 | 198 | Proteoglycans in cancer (FN1 TGFB2 THBS1) |
| 1.0E-03 | 3 | 214 | Regulation of actin cytoskeleton (FGF1 FGF7 FN1) |
| 2.3E-03 | 3 | 291 | Cytokine-cytokine receptor interaction (IL1R1 NGFR TGFB2) |
| 2.3E-03 | 2 | 72 | Melanoma (FGF1 FGF7) |
| 2.8E-03 | 2 | 83 | TGF-beta signaling pathway (TGFB2 THBS1) |
| 3.4E-03 | 2 | 93 | Small cell lung cancer (COL4A1 FN1) |
| 5.6E-03 | 2 | 123 | Platelet activation (COL1A1 COL3A1) |
| 5.6E-03 | 2 | 126 | Osteoclast differentiation (IL1R1 TGFB2) |
| 6.7E-03 | 2 | 150 | Phagosome (COMP THBS1) |
| 6.7E-03 | 2 | 146 | Wnt signaling pathway (DKK1 SFRP2) |
| 6.7E-03 | 2 | 150 | MicroRNAs in cancer (TGFB2 THBS1) |
| 6.7E-03 | 2 | 146 | Breast cancer (FGF1 FGF7) |
| 6.8E-03 | 2 | 154 | Hippo signaling pathway (FGF1 TGFB2) |
| 1.7E-02 | 2 | 255 | HTLV-I infection (IL1R1 TGFB2) |
