# Supplementary material for: Fibrotic Phenotype of Peritumour Mesenteric Adipose Tissue in Human Colon Cancer: A Potential Hallmark of Metastatic Properties
Source: Int J Mol Sci. 2021 Feb 28;22(5):2430. doi: 10.3390/ijms22052430 (PMC7957668; doi:10.3390/ijms22052430)
Supplement: Supplementary file 1 [file ijms-22-02430-s001.zip › Supplementary Table 4.docx]

Supplementary Table 4. Forward and reverse primers.

| **hPLA2G2A**  **Sequence (5'->3')**  Forward primer  Reverse primer | TAGCAACTCGGGGAGCAGAA  GCAGCAGCCTTATCACACTC |
| --- | --- |
| **hFGF7**  **Sequence (5'->3')**  Forward primer  Reverse primer | \| GGCAAAGTAAAAGGGACCCAAG \| \| --- \|   TCCACCCCTTTGATTGCCAC |
| **hCOL1A1**  **Sequence (5'->3')**  Forward primer  Reverse primer | GATTCCAGTTCGAGTATGGCG  TAGGTGATGTTCTGGGAGGC |
| **hSFRP2**  **Sequence (5'->3')**  Forward primer  Reverse primer | GTTTCCCCCAGGACAACGA  TGCAGGCTTCACATACCTTT |
| **hNGFR**  **Sequence (5'->3')**  Forward primer  Reverse primer | CTGCTGCTGTTGCTGCTTC  CACCGCTGTGTGTGTACAGG |
| **hCDH1**  **Sequence (5'->3')**  Forward primer  Reverse primer | CACAGTCACTGACACCAACG  ACGACGTTAGCCTCGTTCTC |
| **LEF1**  **Sequence (5'->3')**  Forward primer  Reverse primer | AGGCTGGTCTGCAAGAGACA  GCTTCGTTTTCCACCTGATGC |
