# Supplementary material for: Fibrotic Phenotype of Peritumour Mesenteric Adipose Tissue in Human Colon Cancer: A Potential Hallmark of Metastatic Properties
Source: Int J Mol Sci. 2021 Feb 28;22(5):2430. doi: 10.3390/ijms22052430 (PMC7957668; doi:10.3390/ijms22052430)
Supplement: Supplementary file 1 [file ijms-22-02430-s001.zip › Supplementary Table 3.docx]

Supplementary Table 3. The table shows KEGG pathways associated with down-regulated genes obtained with ShinyGo.

| **Enrichment FDR** | **Genes in list** | **Total genes** | **Functional Category** |
| --- | --- | --- | --- |
| 7.6E-09 | 9 | 186 | Transcriptional misregulation in cancer (CDKN1B CEBPE FLT3 GZMB HOXA9 HOXA10 ITGB7 MMP9 BAIAP3) |
| 1.9E-07 | 7 | 124 | Cell cycle (CDKN1B CDKN2D PCNA TTK CCNA2 CCNB1 CCND3) |
| 7.0E-07 | 9 | 353 | PI3K-Akt signaling pathway (CDKN1B FLT3 GNG7 ITGB7 MYB PIK3CD DDIT4 CCND3 CD19) |
| 1.2E-06 | 10 | 523 | Pathways in cancer (RASGRP2 CDKN1B FLT3 GLI1 GNG7 MMP9 LEF1 PIK3CD RAC2 CCND3) |
| 5.3E-06 | 7 | 230 | Ras signaling pathway (RASA4 RASGRP2 FLT3 GNG7 PIK3CD RAC2 RASGRF2) |
| 3.8E-05 | 7 | 318 | Human papillomavirus infection (CDKN1B ITGB7 LFNG MFNG PIK3CD CCNA2 CCND3) |
| 9.2E-05 | 5 | 143 | Hepatitis B (CDKN1B MMP9 PCNA PIK3CD CCNA2) |
| 9.2E-05 | 4 | 66 | Acute myeloid leukemia (CEBPE FLT3 LEF1 PIK3CD) |
| 1.0E-04 | 4 | 70 | B cell receptor signaling pathway (PIK3CD RAC2 CARD11 CD19) |
| 1.2E-04 | 5 | 159 | Cellular senescence (PIK3CD MAP2K6 CCNA2 CCNB1 CCND3) |
| 2.2E-04 | 5 | 183 | Chemokine signaling pathway (RASGRP2 CCR7 GNG7 PIK3CD RAC2) |
| 2.7E-04 | 4 | 97 | Prostate cancer (CDKN1B MMP9 LEF1 PIK3CD) |
| 2.8E-04 | 5 | 200 | Epstein-Barr virus infection (CDKN1B PIK3CD MAP2K6 CCNA2 CD19) |
| 6.7E-04 | 3 | 48 | Notch signaling pathway (DTX1 LFNG MFNG) |
| 7.0E-04 | 4 | 131 | FoxO signaling pathway (CDKN1B CDKN2D PIK3CD CCNB1) |
| 7.3E-04 | 5 | 255 | HTLV-I infection (MYB PCNA PIK3CD POLD4 CCND3) |
| 7.3E-04 | 4 | 137 | Fluid shear stress and atherosclerosis (MMP9 PIK3CD MAP2K6 RAC2) |
| 8.8E-04 | 4 | 146 | Wnt signaling pathway (MMP7 LEF1 RAC2 CCND3) |
| 9.3E-04 | 4 | 150 | MicroRNAs in cancer (CDKN1B EZH2 MMP9 DDIT4) |
| 1.1E-03 | 5 | 295 | MAPK signaling pathway (RASGRP2 FLT3 MAP2K6 RAC2 RASGRF2) |
| 1.3E-03 | 3 | 68 | Fc epsilon RI signaling pathway (PIK3CD MAP2K6 RAC2) |
| 2.2E-03 | 4 | 199 | Focal adhesion (ITGB7 PIK3CD RAC2 CCND3) |
| 2.2E-03 | 4 | 200 | Viral carcinogenesis (CDKN1B PIK3CD CCNA2 CCND3) |
| 2.2E-03 | 3 | 86 | Colorectal cancer (LEF1 PIK3CD RAC2) |
| 2.3E-03 | 4 | 205 | Rap1 signaling pathway (RASGRP2 PIK3CD MAP2K6 RAC2) |
| 2.6E-03 | 2 | 22 | Other types of O-glycan biosynthesis (LFNG MFNG) |
| 2.6E-03 | 3 | 95 | Endocrine resistance (CDKN1B MMP9 PIK3CD) |
| 2.6E-03 | 2 | 23 | Mismatch repair (PCNA POLD4) |
| 2.6E-03 | 3 | 96 | Progesterone-mediated oocyte maturation |
| 2.6E-03 | 4 | 222 | Human cytomegalovirus infection |
