# Supplementary figures and images for: Fibrotic Phenotype of Peritumour Mesenteric Adipose Tissue in Human Colon Cancer: A Potential Hallmark of Metastatic Properties
Source: Int J Mol Sci. 2021 Feb 28;22(5):2430. doi: 10.3390/ijms22052430 (PMC7957668; doi:10.3390/ijms22052430)

Gene: Mean vs Standard Deviation

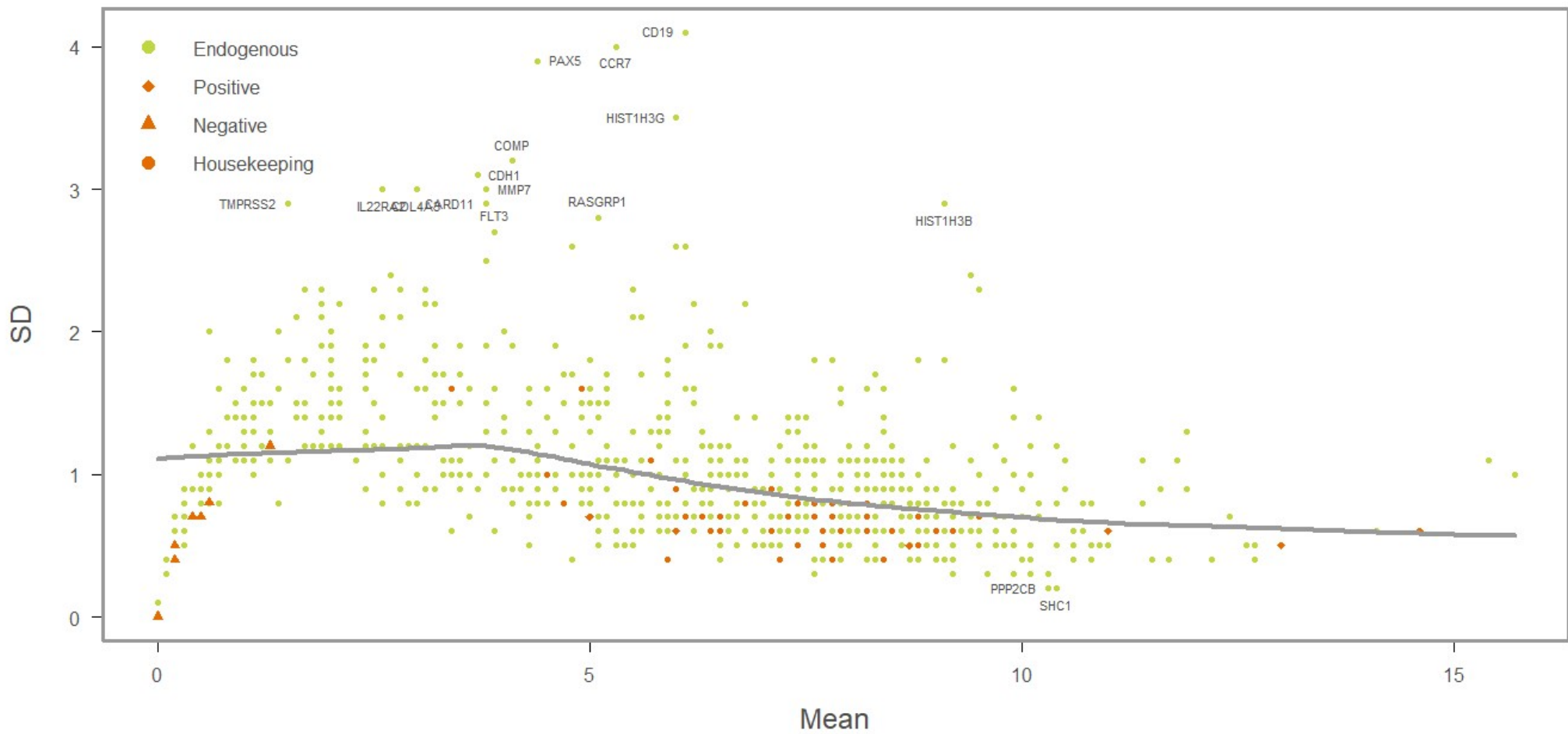

Supplement: Supplementary file 1 [file ijms-22-02430-s001.zip › Supplementary Figure 1.pdf]

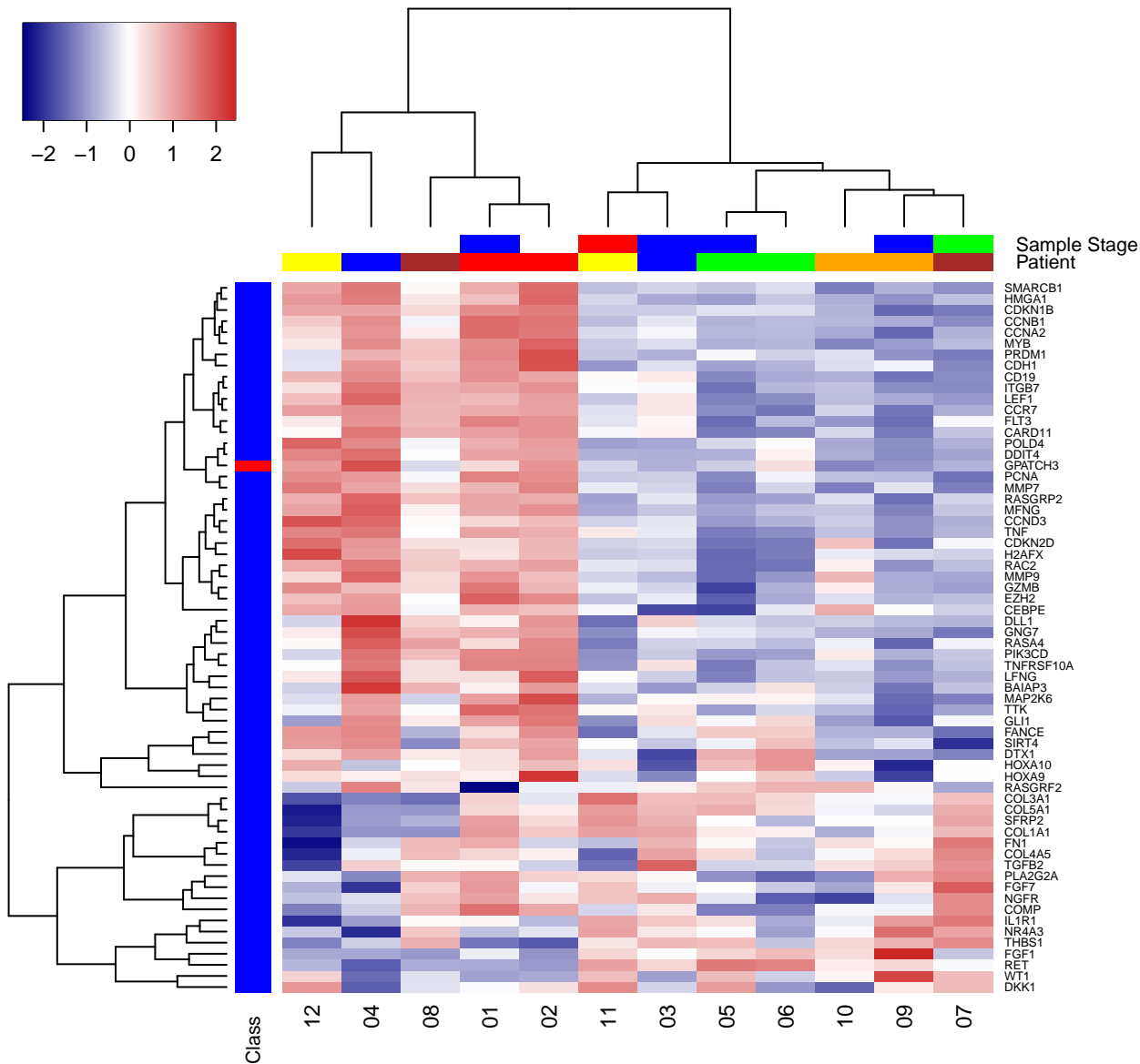

Supplement: Supplementary file 1 [file ijms-22-02430-s001.zip › Supplementary Figure 3.pdf]
